# Inverse relation between serum neurofilament light chain and cognitive function in chronic inflammatory demyelinating polyneuropathy

**DOI:** 10.1007/s00415-025-13179-w

**Published:** 2025-06-03

**Authors:** Rohat Geran, Oliver L. Steiner, Elena Krasivskaya, Ulrike Hannemann, Fabian Klostermann

**Affiliations:** 1 Department of Neurology, Motor and Cognition Group, Charité – Universitätsmedizin Berlin, Freie Universität Berlin and Humboldt-Universität zu Berlin, Campus Benjamin Franklin (CBF), Hindenburgdamm 30, 12203 Berlin, Germany; 2 Berlin School of Mind and Brain, Humboldt-Universität zu Berlin, Berlin, Germany; 3 Institute of Psychology, Humbold-tUniversität zu Berlin, Berlin, Germany

**Keywords:** Chronic inflammatory demyelinating polyneuropathy, Cognition, SNFL, CNS manifestations, Neurodegeneration

## Abstract

**Background:**

Chronic Inflammatory Demyelinating Polyneuropathy (CIDP) is a dysimmune disease primarily targeting the Schwann cell myelin sheath in the peripheral nervous system (PNS), resulting in sensorimotor deficits. Surprisingly, subtle cognitive impairments as well as axonal damage, indicated by elevated serum neurofilament light chain (sNfL), prevail in CIDP. This study investigated whether elevated sNfL is associated with lower cognitive performance in CIDP.

**Methods:**

Thirty-five CIDP patients underwent digital cognitive testing across multiple domains, alongside assessments of sociodemographic, clinical, and sNfL measures. Patients were stratified into low- and high-sNfL groups based on the median value, and clinical variables were compared. Further, general linear models, controlled for clinical and sociodemographic factors, were employed to evaluate the predictive value of sNfL for global and domain-specific cognitive functioning.

**Results:**

Higher sNfL values were associated with worse general cognitive performance (β = −0.31, p = 0.016) and reduced processing speed (β = −0.40, p = 0.008). Patients with increased sNfL levels had a longer disease duration (p = 0.016), also linked to poorer cognitive outcome (β = −0.26, p = 0.045).

**Conclusions:**

In this CIDP cohort, high sNfL levels were associated with reduced cognitive performance and longer disease duration. The findings suggest that sNfL is a clinical meaningful biomarker for the detection and monitoring of central involvement in the course of CIDP, a condition traditionally viewed as purely peripheral.

## Introduction

Chronic Inflammatory Demyelinating Polyneuropathy (CIDP) is a disorder of the peripheral nervous system (PNS), arising from dysimmune processes mainly targeted against Schwann cell epitopes. Slowed or blocked nerval signal conduction due to progressive myelin sheath degradation typically leads to bilateral weakness in proximal and distal limbs, along with sensory impairments [1]. Besides, CIDP variants with either motor or sensory deficits as well as asymmetrical symptom distribution exist [2].

Noteworthy, in addition to the polyneuropathic lead symptoms, a number of non-sensorimotor signs appear to prevail in CIDP. In particular fatigue, depression, retinal and, most surprisingly, subtle cognitive deficits were described [3–7]. The underpinnings of these symptoms remain speculative. For example, it is conceivable that cognitive performance deteriorates as a mediated phenomenon in the context of depressiveness and fatigue in response to the chronic disease burden. Further, an organic basis beyond the classical PNS pathology has been debated with respect to cognitive dysfunctions. For example, it was proposed that dysimmune processes reach central nervous system (CNS) epitopes via a leaky blood–brain barrier [8]. Under the premise of such a nerval background of cognitive CIDP symptoms, serum neurofilament light chain (sNfL) appears an interesting parameter. Elevated sNfL levels are indicative of neuroaxonal damage and have been, amongst others, described in the context of neuroinflammation, both in CIDP and multiple sclerosis (MS) as demyelinating PNS and CNS diseases, respectively [9–13]. Further, as a biomarker of axonal impairment, elevated sNfL levels have been observed in primarily neurodegenerative diseases with cognitive decline [14–16]. So far, it is unknown whether elevated sNfL is associated with low cognitive performance also in CIDP. To close this knowledge gap, we explored this potential link using a broad battery of neuropsychological tests and discussed the results with respect to central involvement in CIDP.

## Methods

### Participants

Thirty-five patients with CIDP were enrolled in the study between August 2021 and February 2024. They were diagnosed according to the EFNS/PNS criteria and on regular maintenance treatment with intravenous immunoglobulins (IvIg) in the neurological outpatient clinic of the Charité, Campus Benjamin Franklin [1]. Among the participants, 26 presented with typical CIDP symptoms, characterized by symmetric proximal, and distal sensorimotor deficits, whereas 9 exhibits CIDP variants. Exclusion criteria included the presence of other neurological or psychiatric conditions that could interfere with cognitive functioning. Patients provided written informed consent to the participation in this study, approved by the ethics committee of the Charité (protocol number EA/165/16).

### Cognition, fatigue and depressive symptoms

Demographic and clinical data were collected exclusively by a trained neuropsychologist (Table 1 for an overview of all cognitive tests and questionnaires used in the neuropsychological assessment). The neurocognitive data were raised using a system for digital psychometric testing (PsyExpert^®^, Wiener Testsystem), a validated standard tool for quantitative and reproducible neurocognitive testing in clinical and research settings.

**Table 1:** Neurocognitive assessment

Domain	Test
Working memory	
Visual	FGT
Auditive	n-back (verbal)
Memory	
Short-term	FGT-recall (short)
Long-term	FGT-recall (long)
Executive functioning	TMT A/B, TOL
Social cognition	TOM
Processing speed/attention	TMT A
Visuoconstruction	VISCO
Divided attention	WAF (cross-modal)
Subjective cognition complaints	FLei
Depression	DESC-I
Fatigue	
Motoric	FSMC motoric score
Cognitive	FSMC cognitive score, WAF (intrinsic alertness)

*FGT* Figuraler Gedächtnistest (figural memory), *TMT A* trail making test A, *TMT B* trail making test B, *TOL* tower of London test, *TOM* theory of mind, *VISCO* visuoconstruction Test, *WAF* (cross-modal) Wiener Aufmerksamkeitstest (cross-modal attention), *FLei* questionnaire for complaints of cognitive disturbances, *DESC-I* Rasch-based depression screening version 1, *FSMC* fatigue scale for motor and cognitive function

Since it took in some cases more than 3 h to run the entire cognitive test battery, the patients performed the tasks in two sessions, in order to keep the strain low and facilitate optimal performances. To control for fatigue levels during testing, a reaction time test (audio, WAF) was performed before and after the assessment. A trained neuropsychologist then categorized levels of cognitive functioning based on the DSM-5 criteria [17]. The classification encompassed four escalating categories based on the absence or presence of impairments in test performances and potential affective or behavioral change (Table 2).

**Table 2:** Cognitive impairment classification criteria

Category	Cognitive performance	Affective/behavioral change
No neurocognitive disorder	None	None
Mild neurocognitive disorder	Reduction in one or more domains (1–2 SD below average)	None or mild
Mild to moderate neurocognitive disorder	Impairment in one or two domains (> 2 SD below average), further reductions possible (1–2 SD below average)	None or mild
Moderate neurocognitive disorder	Impairments in at least two domains (> 2 SD below average); other subfunctions may be mildly impaired	None to moderate

*SD* standard deviation. Criteria for determining the severity of a neuropsychological disorder based on the DSM-5 criteria for assessing the severity of a neurocognitive disorder

### Sensorimotor

Standardized neurological examinations were performed in all patients. Polyneuropathic symptom severity was quantified using the Rasch-built Overall Disability Scale (R-ODS), a standard instrument for the assessment of the sensorimotor impairment in immune-mediated peripheral neuropathies [18]. It provides a composite score from the responses to twenty-four questions concerning fine and gross sensorimotor skills. Answers range from 0 (impossible to perform) to 2 (easy to perform), summing up to a maximum of 48 points indicating absence of any disability, whereas 0 reflects complete disability.

### sNfL analysis

All serum samples were processed and analyzed at the Labor Berlin–Charité Vivantes GmbH. sNfL levels were measured using the Single Molecule Array (SIMOA) technology on the HD-X Analyzer (Quanterix Inc., Billerica, MA, USA) using the commercially available NfL advantage kit [19]. This digital immunoassay is based on two highly specific, non-competitive monoclonal antibodies that can detect single molecules bound to paramagnetic beads and achieve sensitivity down to femtomolar concentrations [20]. Age- and BMI-adjusted sNfL z-scores were calculated through the sNfL Reference App online service (https://shiny.dkfbasel.ch/baselnflreference/), following the method outlined by Benkert et al. [21].

### Statistics

All analyses were performed in R (version 4.4.2). We first examined the association between sNfL levels and key clinical parameters (fatigue, depression, motor symptom severity, disease duration, and IvIg dose). Participants were divided into low and high sNfL groups based on the median sNfL level. The resulting groups were then compared. For normally distributed variables we conducted t-tests, for non-normally distributed variables we used Wilcoxon rank-sum tests. For the total cohort we calculated Pearson’s correlations for normally distributed data and Spearman correlations for non-normally distributed data.

Next, we investigated the relationship between sNfL and cognitive performance. We began by creating a Global Cognition (GC) composite score created by the mean of the standardized multiple cognitive domains. Before fitting regression models, we used the car package to verify the assumptions of multiple regression (linearity, homoscedasticity, normality of residuals) [22]. All assumptions were satisfied except for cross-modal attention, which showed non-normally distributed residuals, and figural long-term memory, which was characterized by heteroskedasticity, indicated by the Breusch–Pagan test. We then fitted generalized linear models (GLMs) with GC as the outcome and sNfL, age, education, depression, fatigue, test-fatigue, disease duration, and R-ODS scores as predictors. This procedure was further repeated for each cognitive subdomain. Because cross-modal attention violated the normality assumption of the residuals, we used the sandwich package to obtain robust standard errors and to address residual non-normality [23]. For the figural long-term memory model, we decided to fit a general least squares (GLS) model as this approach is more robust for heteroskedastic data [24]. For both models, significance was assumed at α ≤ 0.05.

After building linear regression models, we estimated the likelihood that the selected significant predictors would be included in the “true” model for predicting cognitive performance using Bayesian Model Averaging (BMA). In BMA, unlike linear regression, not a single “best” model is selected, but all possible combinations of predictors are computed and assigned with a posterior probability. This posterior probability measures how each model fits the current data. For example, in the present scenario with 9 predictors, 512 models (2^9^) are calculated and rated with a posterior probability. By summing the normalized posterior probabilities of all models that include a given predictor (e. g. depression), one obtains the posterior inclusion probability (PIP) of that predictor in the “true” outcome model [25]. In general, a PIP above 0.5 is considered meaningful, whereas a PIP above 0.9 is regarded as strong evidence that the predictor is part of the “true” model [26]. Here, we implemented BMA using the BMS package in R, including all predictor combinations in the analysis (mprior =“uniform”) [27]. Since there is no prior data on the influence of predictors on cognition in CIDP, we set the prior distribution of predictor influence to the default setting (g = “UIP”).

Finally, to compare differences among cognitive categories, we used a one-way ANOVA with depression and fatigue as covariates and examined post-hoc results using Tukey’s post hoc test.

## Results

Demographic and clinical characteristics of the presented cohort are shown in Table 3. Our cohort included thirty-five patients with CIDP, 10 (28.6%) of whom were female, with a mean age of 62.5 years (SD: 12.6) at inclusion. Twenty-six patients (74.3%) were diagnosed with typical CIDP, while the remaining nine patients had CIDP variants (multifocal CIDP *n* = 3, sensory CIDP *n* = 1, distal CIDP *n* = 5). The disease duration was highly variable with a median of 55.0 months (IQR: 90.0). The median IvIg dose was 80 g (IQR: 20.0), with treatment intervals from 21 to 56 days (median: 28). No patient received additional immunotherapy (e.g. rituximab). sNfL levels were 18.4 pg/mL on average (SD: 23.1; median: 11.9; IQR: 7.0). The clinical and demographic characteristics correspond well with previously published CIDP cohorts, which supports the representativeness of our population [9, 28].

**Table 3:** Main demographic and clinical characteristics

	Overall (N = 35) mean ± SD (median; range; IQR)
Age (y)	62.5 ± 12.6 (63.0; 32.0–87.0; 12.0)
Sex, (female), n (%)	10 (28.6)
Age at diagnosis (y)	56.2 ± 11.7 (59.0; 31.0–76.0; 14.0)
Disease duration (m)	74.5 ± 69.8 (55.0; 3.0–266.0; 90.0)
Patients with typical CIDP, *n* (%)	26 (74.3)
sNfL value, (pg/ml)	18.4 ± 23.1 (11.9; 4.1–131.0; 7.0)
sNfL Z-score	0.2 ± 1.3 (0.1; −2.2–3.2; 1.5)
BMI, (kg/m^2^)	27.1 ± 4.4 (27.7; 18.4–36.0; 4.6)
R_ODS	35.4 ± 10.4 (38.0; 9.0–48.0; 13.2)
DESC-I	7.4 ± 8.5 (5.0; 0.0–33.0; 7.5)
FSMC total	55.0 ± 25.0 (51.5; 0.0–100.0; 39.8)
FSMC cog score	24.9 ± 12.4 (22.5; 0.0–50.0; 21.2)
FSMC mot score	29.6 ± 12.7 (30.0; 0.0–50.0; 19.2)
IvIg dosage, (g)	84.1 ± 16.5 (80.0; 50.0–110.0; 20.0)
IvIg interval length, (d)	33.4 ± 8.2 (28.0; 21.0–56.0; 14.0)

*BMI* body mass index, *CIDP* chronic inflammatory demyelinating polyneuropathy, *DESC-I* Rasch-based depression screening version 1, *FSMC* fatigue scale for motor and cognitive functions, *IQR* interquartile range, *IvIg* dosage total dosage of intravenous immunoglobulin administered, *IvIg* interval length interval between intravenous immunoglobulin treatments in days, *R_ODS* Rasch-built overall disability scale, *SD* standard deviation, *sNfL* serum neurofilament light chain

### sNfL vs clinical variables

Participants were divided into low and high sNfL groups based on the median sNfL level (low level group: 8.59 ± 2.96 pg/mL, high level group: 28.81 ± 29.95 pg/mL; W = 3.89, *p* < 0.001). Details are summarized in Table 4. There was no significant difference in sex distribution (p- > 0.05). The high sNfL group tended to be older (66.88 ± 13.57 vs. 58.33 ± 10.28 years; W = 1.68, *p* = 0.096) and had a significantly longer time since diagnosis (99.06 ± 70.98 vs. 51.39 ± 61.93 months; W = 2.42, *p* = 0.016). Educational level, R_ODS, depression, fatigue (cognitive + motoric), IvIg dose, IvIg interval length, did not differ significantly between the groups (*p* > 0.05).

**Table 4:** sNfL vs clinical variables

Variable	Groupe Comparison			Correlations	
	sNf-z-low (n = 18) (mean ± SD)	sNfL-z-high (n = 17) (mean ± SD)	*p*	*r*	*p*
sNfL (pg/mL)	8.59 ± 2.96	28.81 ± 29.95	< 0.001	0.853	< 0.001
Age (y)	58.33 ± 10.28	66.88 ± 13.57	0.096	0.43	0.009
Sex (female)	5	5	1.000		
Education	3.72 ± 0.96	3.65 ± 0.93	0.843	−0.07	0.689
Time since diagnosis (m)	51.39 ± 61.93	99.06 ± 70.98	0.016	0.34	0.048
DESC-I	8.61 ± 9.91	6.12 ± 6.88	0.921	−0.04	0.701
FSMC (total)	49.67 ± 26.08	61.06 ± 22.98	0.254	0.08	0.663
R_ODS	37.23 ± 10.33	33.40 ± 10.44	0.273	−0.23	0.198
IvIg dosage (g)	87.78 ± 16.02	75.00 ± 23.05	0.099	−0.30	0.084
IvIg interval length (d)	33.83 ± 8.05	32.94 ± 8.49	0.753	−0.10	0.763

*CSF* cell cell count in the cerebrospinal fluid, *DESC-I* Rasch-based depression screening version 1, *FSMC* fatigue scale for motor and cognitive functions, *IvIg* dosage total dosage of intravenous immunoglobulin administered, *IvIg* interval length (d) interval between intravenous immunoglobulin treatments in days, *R_ODS* Rasch-built overall disability scale

P-values for mean differences between groups were determined using two-sided t-tests for normally distributed data and the Wilcoxon rank-sum test for non-normally distributed data. The threshold for assuming significance was set at α ≤ 0.05. Correlations (r) were calculated with Pearson’s correlation for normally distributed variables and Spearman’s rank correlation for non-normally distributed variables.

sNfL was positively associated with age (r = 0.43, *p* = 0.010) and with time since diagnosis (r = 0.34, *p* = 0.048). Correlations with other clinical parameters were not found (p > 0.05).

### sNfL vs cognition

Within the GLM and GLS model framework, several factors emerged as significant predictors for cognitive performance (Table 5). Higher education consistently showed a positive association with better cognitive performance. Individuals with higher education demonstrated better GC (β = 0.41, 95% CI 0.159 to 0.658, *p* = 0.004), improved visuoconstruction abilities (β = 0.47, 95% CI 0.219 to 0.729, *p* = 0.001), as well as enhanced short-term (β = 0.36, 95% CI 0.049 to 0.673, *p* = 0.032) and better auditive working memory performance (β = 0.426, 95% CI 0.028 to 0.768, *p* = 0.022). Higher sNfL levels were associated with reduced GC (β = −0.31, 95% CI −0.542 to −0.076, *p* = 0.016), impaired visuoconstruction (β = −0.28, 95% CI −0.521 to −0.043, *p* = 0.029), slower processing speed/attention as indicated by worse performance on the Trail Making Test A (β = −0.40, 95% CI −0.680 to −0.128, *p* = 0.008), and with a trend to lowered cross-modal attention (β = −0.35, 95% CI −0.689 to −0.016, *p* = 0.059). Longer disease duration corresponded to subtle declines in GC (β = −0.26, 95% CI −0.501 to −0.018, *p* = 0.045). Female participants outperformed male participants on the TOM test (β = 0.37, 95% CI 0.036 to 0.709, *p* = 0.040). Test-fatigue was associated with reduced processing speed/attention (β = 0.35, 95% CI 0.0664 to 0.636, *p* = 0.024). All other predictors were not significant in the performed models (*p* > 0.05).

In contrast, subjective cognitive complaints and their components (attention, memory, and executive facets) were predominantly influenced by depression and fatigue rather than by sNfL levels. Higher depression scores were linked to more severe overall subjective complaints (β = 0.38, 95% CI 0.133 to 0.625, *p* = 0.006) and greater problems in attention (β = 0.43, 95% CI 0.160 to 0.701, *p* = 0.005) and executive functioning (β = 0.39, 95% CI 0.136 to 0.646, *p* = 0.006). Fatigue also contributed to more reported subjective issues, notably general complaints (β = 0.31, 95% CI 0.046 to 0.574, *p* = 0.030) and attention-related complaints (β = 0.34, 95% CI 0.048 to 0.629, *p* = 0.031). Test-fatigue was related to complaints about executive functioning (β = −0.30, 95% CI −0.504 to −0.086, *p* = 0.010). Importantly, no significant associations were found between sNfL and subjective cognitive complaints (p > 0.05). Education was linked with fewer subjective complaints about memory impairments (β = −0.26, 95% CI −0.500 to −0.015, *p* = 0.047).

The additional BMA analysis validated the initial findings (Table 5), identifying predictors that were likely included in the true model for various cognitive domains and subjective complaints. Education was likely to be included as a predictor for cognition in CG (PIP = 0.993, PostMean = 0.475, 95% CI 0.236 to 0.714), visuoconstruction (PIP = 0.996, PostMean = 0.712, 95% CI 0.270 to 0.780), long-term figural memory (PIP = 0.789, PostMean = 0.327, 95% CI −0.236 to 1.660), short-term figural memory (PIP = 0.843, PostMean = 0.327, 95% CI −0.056 to 0.711), and auditive working memory (PIP = 0.922, PostMean = 0.403, 95% CI 0.047 to 0.759). sNfL was also likely included in the true model as a predictor for CG (PIP = 0.934, PostMean = −0.321, 95% CI −0.591 to −0.050), visuoconstruction (PIP = 0.896, PostMean = −0.301, 95% CI −0.597 to −0.005), processing speed/attention (PIP = 0.964, PostMean = −0.425, 95% CI −0.722 to −0.128) and cross-modal attention (PIP = 0.827, PostMean = –0.331, 95% CI –0.722 to –0.730). Disease duration was likely included as a predictor for cognition in CG, showing a negative association (PIP = 0.799, PostMean = −0.227, 95% CI −0.523 to 0.070). Age was likely included as a predictor for short-term figural memory (PIP = 0.838, PostMean = −0.353, 95% CI −0.779 to 0.072) and auditive working memory (figural; PIP = 0.879, PostMean = −0.412, 95% CI −0.834 to 0.009). Test-fatigue was likely included as a predictor for processing speed/attention (PIP = 0.826, PostMean = −0.205, 95% CI −0.456 to 0.046), subjective overall complaints (PIP = 0.826, PostMean = −0.205, 95% CI −0.456 to 0.045), and subjective complaints about executive functioning (PIP = 0.953, PostMean = −0.312, 95% CI −0.549 to −0.074).

**Fig. 1:**
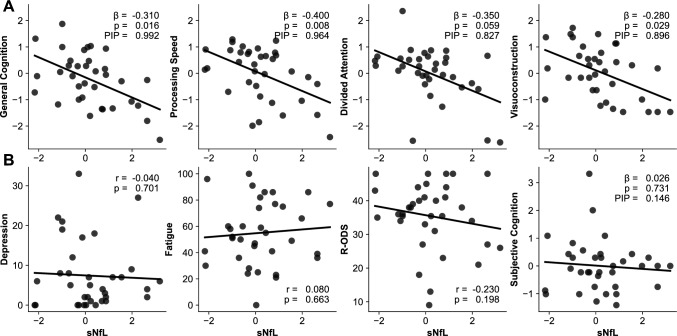
Relationship between sNfL scores and **A** cognitive performance (expressed as z-scores) and **B** various clinical parameters. For all cognitive scores, the presented β and *p* values are derived from GLM models including age, depression, fatigue, disease duration, and motor symptoms as covariates. *R-ODS* Rasch-built overall disability scale, *GLM* generalized linear model, *sNfL* serum neurofilament light chain corrected for age and BMI, *PIP* posterior inclusion probability, *BMA* Bayesian model averaging

Depression and fatigue were consistently likely to be included in models predicting subjective complaints across attention, memory, and executive functioning. Both depression and fatigue showed high inclusion probabilities (PIP = 0.989 to 0.995 and 0.907 to 0.945, respectively). The sex of participants was likely included in the model to predict social cognition (PIP = 0.783, PostMean = 0.288, 95% CI −0.100 to 0.676). All other parameters were not likely to be model predictors (PIP ≤ 0.75) (Table 5) (Fig. 1).

**Table 5:** Predicting cognition

Test	Predictor	β	*p*	PIP
General	Education	**0.41**	**0.004**	**0.992**
	sNfL	**−0.31**	**0.016**	**0.934**
	Disease duration	**−0.26**	**0.045**	**0.800**
	Depression	0.00	1.000	0.112
	Fatigue	−0.20	0.230	0.162
	Test-fatigue	0.08	0.534	0.205
	Age	−0.13	0.405	0.162
	Sex	0.12	0.375	0.179
Objective cognitive subdomains vs sNfL				
FGT	sNfL	−0.16	0.349	0.224
n-back (verbal)		−0.07	0.683	0.181
FGT-recall (short)		−0.10	0.497	0.216
FGT-recall (long)		−0.29	0.122	0.532
TMT A/B		−0.13	0.115	0.349
TOL		−0.17	0.344	0.340
TOM		−0.20	0.204	0.436
TMT A		**−0.40**	**0.008**	**0.964**
VISCO		**−0.28**	**0.029**	**0.896**
WAF (cross-modal)		−0.35	0.059	0.827
Subjective cognitive estimation vs sNfL and depression and fatigue				
FLei (total)	sNfL	0.03	0.731	0.146
	Depression	**0.38**	**0.006**	**0.990**
	Fatigue	**0.31**	**0.030**	**0.907**

Bold values mark significant predictors with above chance of 75% to be included

*FGT* Figuraler Gedächtnistest, *TMT A* trail making test A, *TMT B* trail making test B, *TOL* tower of London test, *VISCO* visuoconstruction test, *WAF* (cross-modal) Wiener Aufmerksamkeitstest (cross-modal), *FLei* questionnaire for complaints of cognitive disturbances, *DESC-I* Rasch-based depression screening version 1, *FSMC* fatigue scale for motor and cognitive functions

β standardized beta coefficients from models. PIP posterior inclusion probability derived from Bayesian model averaging.

### Cognitive state

**Fig. 2:**
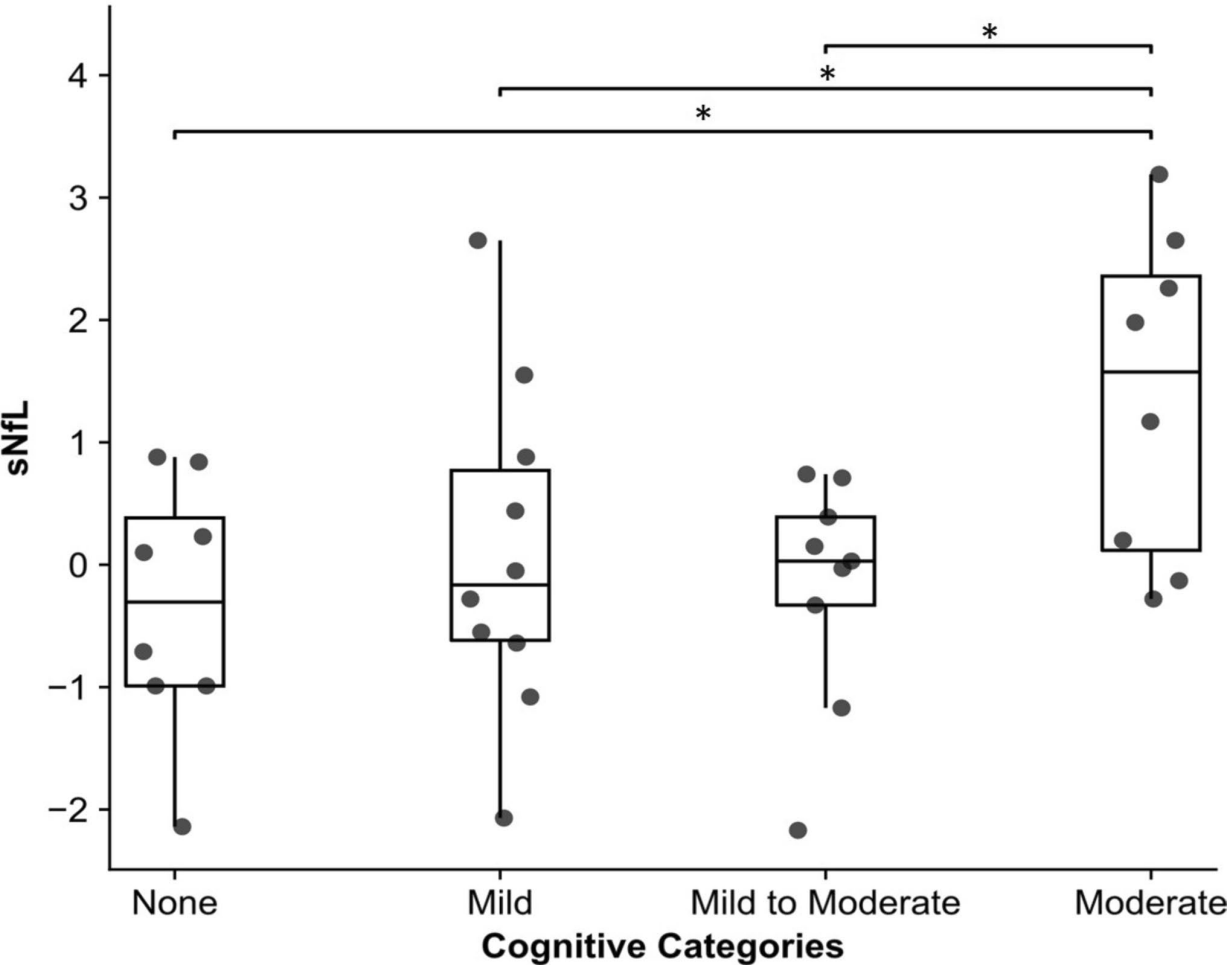
Comparison of sNfL z-scores across DSM-5–based cognitive impairment categories in CIDP patients. Categories include no/mild/mild-to-moderate/moderate neurocognitive disorder. sNfL values are presented as age- and BMI-adjusted z-scores. Error bars represent the standard error. *p ≤ 0.05, based on one-way ANOVA with Tukey’s post hoc test

Using an ANOVA approach with Tukey’s post hoc test, there was a significant difference in sNfL levels across cognitive impairment groups. Patients in the moderate group had significantly higher sNfL levels compared to those without cognitive impairment (mean = −0.429, standard error (SE) = 0.431, *p* = 0.016), those with mild impairment (mean = −0.042, SE = 0.380, *p* = 0.043), and those with mild-to-moderate impairment (mean = −0.255, SE = 0.394, *p* = 0.020). No other pairwise comparisons were significant (all *p* > 0.05) (see Fig. 2).

## Discussion

In the studied CIDP group, global cognition correlated inversely with sNfL levels, i.e., the lower patients generally performed in the cognitive test battery, the higher their sNfL levels were. Further, those patients who were clinically categorized in the group with lowest cognitive performance, equating to a moderate cognitive deficit, showed the highest sNfL levels. Additionally, on the task level of single tasks sNfL was with a high degree of certainty a predictor of TMT A performance, reflecting processing speed and attention. Interestingly, the determined sensorimotor disease severity, fatigue and depressiveness neither predicted the variance of cognitive performances, nor did they differ between patients with high vs low sNfL levels. Thus, the findings suggest that sNfL can be used as a biomarker for cognitive dysfunction in CIDP, regardless of the further disease burden.

Cognitive deficits in CIDP were repeatedly reported in recent studies, but their underpinnings and embedding in the clinical phenotype remain unknown [6–8]. In this regard, the observed association of sNfL and cognitive performance is of interest. In general, elevated sNfL levels are increasingly recognized as an unspecific marker of axonal damage. Accordingly, they have been detected in neurodegenerative conditions with cognitive decline, reflecting incremental neuronal loss over time [14, 29, 30]. However, sNfL elevation has also been reported in neuroinflammatory entities, such as CIDP and MS [10–12, 31]. Here, sNfL did not only inversely correlate with cognitive performance, but patients with high sNfL levels also had longer disease durations compared to those with lower levels. Thus, the presented findings are well compatible with the idea of a pathological process in the long-term course of CIDP, reflected by increased sNfL. This, impacting on cognitive functioning, probably involves the CNS.

The inverse relation of sNfL and TMT A performance in the present patient cohort deserve a particular mention, since it points to associations with attentional processing and cognitive speed. Notably, the latter has been reported in previous CIDP studies and it was repeatedly described in MS [7, 8, 32, 33]. Already in early MS stages cognitive slowing can emerge without physical disability and high sNfL values were shown to be associated with this symptom as well as with neuroaxonal damage in this centrally defined inflammatory CNS disease [12, 34–36]. In analogy to this, similar deficits in CIDP could reflect minor axonal CNS affection as a result of chronic neuroinflammation. As a possible pathomechanism for such central involvement, some permeability of the blood–brain barrier for dysimmune processes were previously discussed [8]. In this view, CIDP and MS can be conceived as embedded in a spectrum of variable PNS-to-CNS inflammatory affection with completely mixed, yet seldom cases of Combined Central and Peripheral Demyelination (CCPD) in its middle [37, 38]. Thus, processing speed might be a cognitive function particularly vulnerable to CIDP pathology, principally similar to alterations described in MS.

Concerning non-sensorimotor features, fatigue is probably most recognized and has been repeatedly described in CIDP [4, 39]. In the current context, it is noteworthy that this symptom, also comprising cognitive fatigue, was neither predictive of the tested cognitive performances, nor did it correlate with sNfL. Therefore, fatigue in CIDP could, e.g., entail a closer relation to the primarily inflammatory disease pathology with its demyelinating consequences rather than to the axonal damage arising in the long-term course of the disease [40]. Absence of a relation to cognitive performance and sNfL was also obtained for depressiveness and sensorimotor disease severity as expressed by the R_ODS. Thus, these disease aspects could also depend more on the inflammatory than on the degenerative CIDP pathology.

Our study has several limitations. Firstly, we used a cross-sectional approach, which does not allow to evaluate the prognostic value of sNfL for the cognitive development of patients. To assess this, longitudinal studies with repeated cognitive and sNfL measurements throughout the course of CIDP would be needed. Secondly, the current CIDP cohort was recruited from a single specialized center, so that a selection bias towards complex or treatment-resistant cases cannot be excluded. However, such an effect should be small, because first-line infusion therapies for CIDP are predominantly provided by tertiary hospitals with according outpatient facilities in Germany. Further, investigated CIDP cohorts would ideally have a narrow age range, given the age-dependency of sNfL and a possible mediation of both sNfL increase and cognitive decline by aging. This, however, is practically difficult for the relatively low prevalence of the disease. Therefore, we chose to statistically control for this possibility and used well established age and BMI-adjusted sNfL z-scores [21]. Moreover, we included age as a covariate in the prediction models for cognition. Finally, while the current study points to CNS involvement in CIDP patients, reflected by high sNfL together with low cognitive performance, the underpinnings of this nexus should be directly addressed by brain imaging methods in future studies.

In sum, this study suggests that sNfL can be used as a biomarker for subtle central pathology, which can occur in the course of CIDP. Specifically, monitoring of sNfL could be instrumental in identifying patients at risk of cognitive decline and, thus, in supporting a holistic disease management. Future longitudinal studies implying larger patient cohorts would help to confirm this new approach in a disease traditionally viewed as purely peripheral.

## Data Availability

The anonymized source data of this study are available upon request.
